# Using systems thinking to understand the scale-up and sustainability of health innovation: a case study of seasonal malaria chemoprevention processes in Burkina Faso

**DOI:** 10.1186/s12889-023-16729-x

**Published:** 2023-10-02

**Authors:** Marietou Niang, Marie-Pierre Gagnon, Sophie Dupéré

**Affiliations:** 1 Department of Psychosociology and Social Work, Université Québec À Rimouski (UQAR), Campus de Lévis, Québec, Canada; 2 Faculty of Nursing Science, Université Laval, Québec, QC Canada

**Keywords:** Scale-up, Sustainability, Innovation, Africa, Burkina Faso, Malaria, Seasonal malaria chemoprevention, Systems thinking

## Abstract

**Background:**

Scale-up and sustainability are often studied separately, with few studies examining the interdependencies between these two processes and the implementation contexts of innovations towards malaria prevention and control. Researchers and implementers offer much more attention to the content of innovations, as they focus on the technological dimensions and the conditions for expansion. Researchers have often considered innovation a linear sequence in which scaling up and sustainability represented the last stages. Using systems thinking in this manuscript, we analyze complex scaling and sustainability processes through adopting and implementing seasonal malaria chemoprevention (SMC) in Burkina Faso from 2014 to 2018.

**Methods:**

We conducted a qualitative case study involving 141 retrospective secondary data (administrative, press, scientific, tools and registries, and verbatim) spanning from 2012 to 2018. We complemented these data with primary data collected between February and March 2018 in the form of 15 personal semi-structured interviews with SMC stakeholders and non-participant observations. Processual analysis permitted us to conceptualize scale-up and sustainability processes over time according to different vertical and horizontal levels of analysis and their interconnections.

**Results:**

Our results indicated six internal and external determinants of SMC that may negatively or positively influence its scale-up and sustainability. These determinants are effectiveness, monitoring and evaluation systems, resources (financial, material, and human), leadership and governance, adaptation to the local context, and other external elements. Our results revealed that donors and implementing actors prioritized financial resources over other determinants. In contrast, our study clearly showed that the sustainability of the innovation, as well as its scaling up, depends significantly on the consideration of the interconnectedness of the determinants. Each determinant can concurrently constitute an opportunity and a challenge for the success of the innovation.

**Conclusion:**

Our findings highlight the usefulness of the systemic perspective to consider all contexts (international, national, subnational, and local) to achieve large-scale improvements in the quality, equity, and effectiveness of global health interventions. Thus, complex and systems thinking have made it possible to observe emergent and dynamic innovation behaviors and the dynamics particular to sustainability and scaling up processes.

**Supplementary Information:**

The online version contains supplementary material available at 10.1186/s12889-023-16729-x.

## Introduction

Since 2012, the World Health Organization (WHO), based on seven experimental studies, has recommended seasonal malaria chemoprevention (SMC) as an innovative preventive strategy in highly seasonal malaria transmission areas [1]. The SMC intervention consists of a monthly and intermittent administration of a complete Sulfadoxine-Pyrimethamine and Amodiaquine (SP + AQ) to eligible children aged three to 59 months for up to four months during the high transmission season. It aims to maintain therapeutic blood levels throughout the high transmission season to prevent malaria. Experimental studies found that SMC could result in a 75% reduction in malaria cases, and the intervention is low in cost, safe, and feasible [2, 3].

Since 2014, Burkina Faso has introduced SMC to fight malaria, and health officials have progressively scaled it across the country. There is some evidence of the effectiveness of SMC in routine conditions [4–6]. One of these studies [6] conducted in the pilot health district of Kaya (Burkina Faso) in 2014–2015 reported a 51% (95% CI = [0.24–0.99]) and 62% (95% CI = [0.29–0.52]) protective effect of SMC on periodic prevalence. However, the sustainability of this type of intervention is a challenge, especially in the long run, because it could interfere with the development of natural immunity and likely increase drug resistance in cases of large-scale use [7, 8]. Additionally, the complexity of events and contexts makes the durability and integration of preventive intervention a difficult challenge for organizations and health systems [9, 10]. Moreover, the scaling up and sustainability of malaria-related interventions do not depend solely on the long-term effectiveness of antimalarial drugs. There are other critical factors such as the adherence and compliance of community members, availability and cost of drugs, involvement and capacity of the public health system, integration of the intervention in the community-based program [7, 11, 12] and several contextual factors such as insecurity or environmental changes [13, 14].

In general, few scientific studies focus on the processes of scaling up or sustaining innovations and their determinants of success and failure. Researchers and implementers give much more attention to the content of innovations, including the availability of resources or antimalarial drugs. This study uses systems thinking to understand SMC’s scaling up and sustaining evolution in Burkina Faso from its adoption in 2014 to the end of 2018. We aim to fill some knowledge gaps by illustrating the dynamic interplay between the determinants of the scaling up and sustainability of SMC and the interconnectedness between determinants and the contexts of implementation.

## Theoretical perspectives: complex and systems thinking

**Fig. 1:**
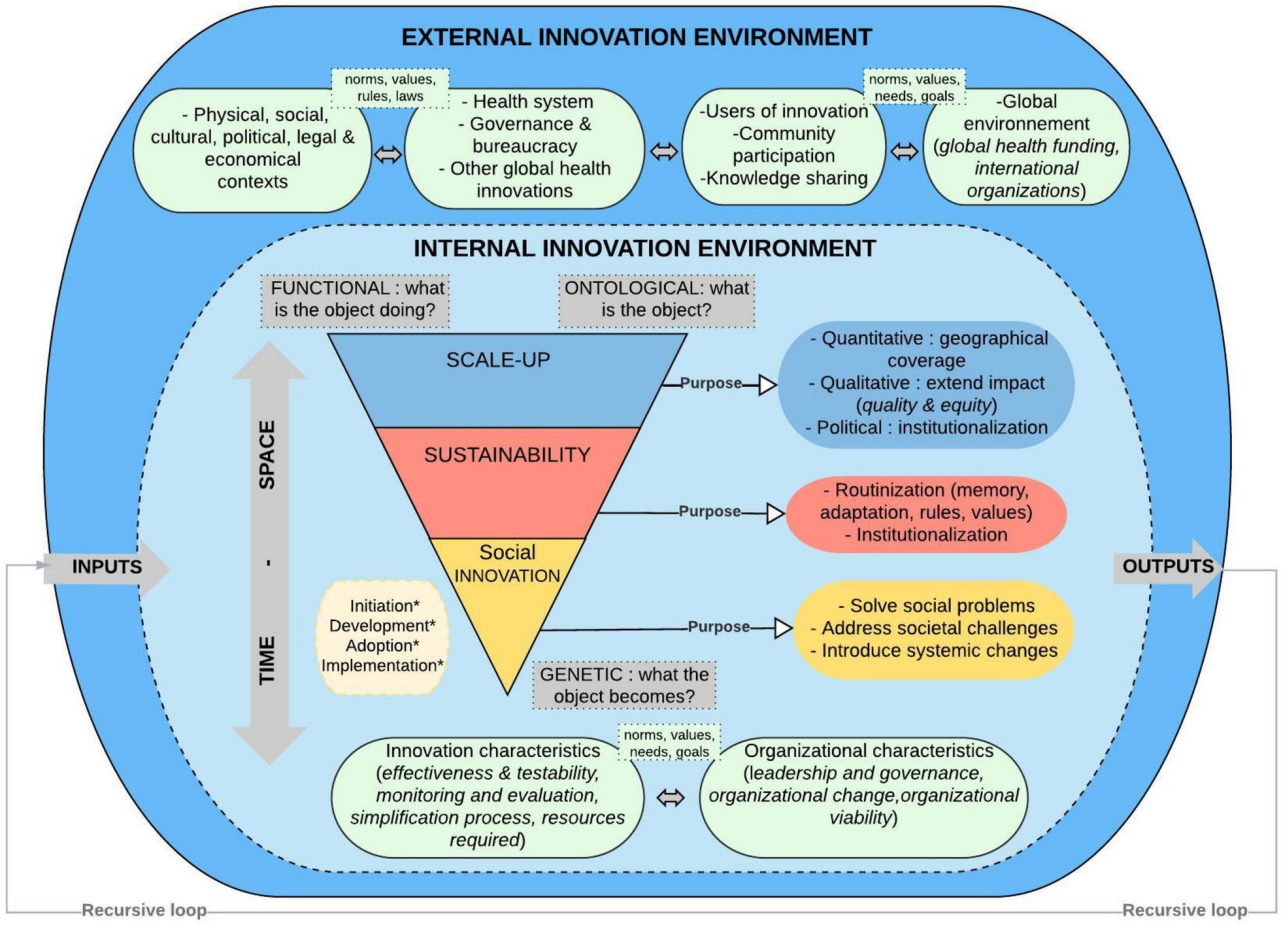
An integrative conceptual framework of scale-up and sustainability [15]. Notes: The grey colour represents the elements coming from the systems thinking approach presented in the manuscript background. The other boxes represent the different elements found in our literature review. It must be understood that all the elements are interconnected and influence each other. The elements with * presented in the dotted box represent other processes that can take place during the scale-up and sustainability processes

The conceptual framework presented in Fig. 1 was developed in a previous article [15] and guided this research. We conceived this framework by using a narrative review on the scale-up and sustainability of innovation in global health integrated with the general systems theory (modelling theory) [16]. This theory is rooted in a complex and systemic paradigm that allows a symbolic representation of a system. It aims to model a complex perceived phenomenon and grapple with its intelligibility. It conceives of a system like an object in an environment and endows it purposefully. A system is comprised of several closely interlinked parts, forming a complex structure with permeable boundaries that shift between the entities and their environment. Innovation processes are part of this understanding of self-organized systems with emergent properties. Their internal elements are interrelated and evolve with the external environment.

In our framework, SMC is considered an innovation system, and scaling up and sustainability processes are its subsystems, all of which have purposes. Innovation, understood in the social paradigm, aims to solve social problems, address societal challenges, or introduce systemic changes. *Sustainability* is a dynamic process that aims to achieve the long-term viability of an innovation, without external funding, through routinization and institutionalization in existing organizations or institutions [17, 18]. We conceptualized scale-up into three broad, complementary dimensions [15]. The *quantitative dimension* focuses on geographic expansion [19]. The *qualitative dimension* refers to extending the innovation's impact equitably and sustainably [20] while offering attention to hard-to-reach populations to ensure equity. The *policy dimension* refers to institutionalizing or integrating a proven innovation into the existing health system, focusing on institutional capacity building and sustainability [21].

All innovation processes are interconnected in time and space, considering the historical, territorial, and institutional contexts in which SMC fits and unfolds. Also, we consider these processes as an arrangement of interlocking structures and processes [22] that are locally and socially organized actions [23]. An open system is characterized through interactions with its environment through exchanges (material, energetic or informational) and by inputs and outputs [16]. Thus, working dynamically, an open system interacts at different levels: the system itself (between its internal parts or subsystems), its immediate environment, and the surrounding or super-system environment. SMC's functioning in its internal and external environments is an essential point for this study.

## Methods

### Sampling methods

We adopted a maximum variation sampling [24] for the broadest range of perspectives possible about SMC's scale-up and sustainability processes. This sampling method permits external or contrast diversification within the studied case. We recruited stakeholders at different scales of innovation implementation; these stakeholders were national (at the level of ministries and international organizations), regional (regional health directorate, supporting organizations), or local (lead organizations, user organizations, or individuals).

### Data collection

Data collection combined various methods: documentary analysis, individual interviews, and observation (see Additional file 1 for details). We used two data sources. One is secondary, i.e., data collected by local or international organizations working towards malaria prevention and control or by the research program: Interventions to Improve Maternal, Newborn, and Child Health in Mali and Burkina Faso (ISMEA). The other source is primary, i.e., data collected by the first author of this article. The documentary analysis (*n* = 141) included administrative and scientific data (*n* = 37), tools and registries (*n* = 36), press articles (*n* = 2), and verbatim quotations (*n* = 66) from interviews collected in three sanitary districts (Boulsa, Fada, and Tougan) by the ISMEA program. We selected these documents according to specific criteria, notably their authenticity (author, source, purpose), source (primary or secondary), and relevance (links with SMC and pertinence to answering research questions). These data covered innovation emergence, adoption, and implementation periods from 2012 to 2018.

In addition, between February and March 2018, the first author (MN) carried out a peripheral observation [25] in Burkina Faso for 45 days. Then, she conducted 15 individual interviews with different SMC stakeholders: central level (*n* = 9), peripheral level (*n* = 2), and some local researchers (*n* = 3). These participants have occupied different functions in the innovation's planning, implementation, or research since 2014 (*n* = 9) and others since 2015 (*n* = 2), 2016 (*n* = 2) and 2017 (*n* = 2). We used an interview guide during interviews, developed in coevolution with data collection, and adjusted for the participants.

**Fig. 2:**
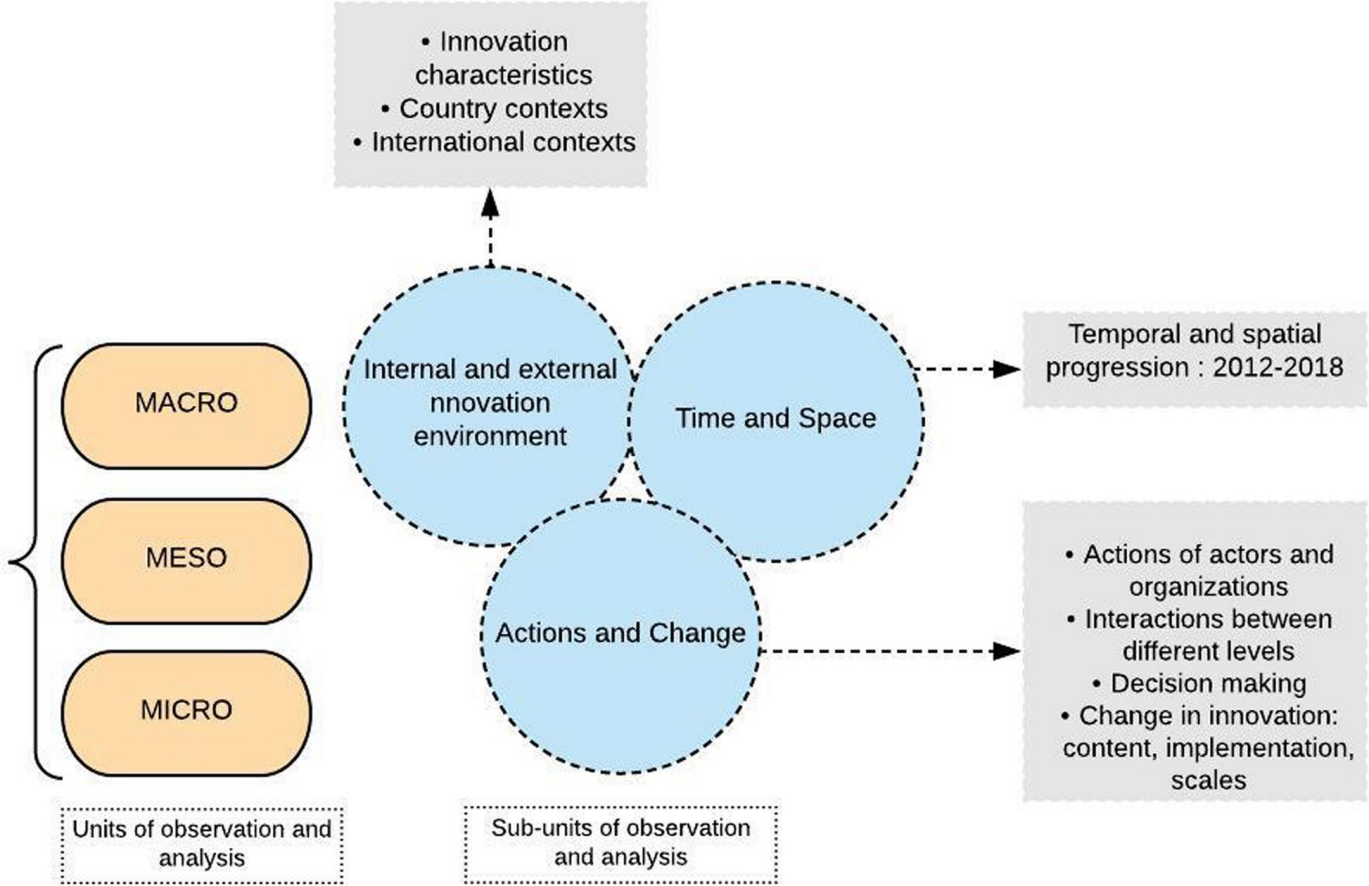
Units and sub-units of observation and analysis

The triangulation of methods offers the advantage of each method's specific strengths. Data collected were different: processual, pluralistic, historical, and contextual [22]. We considered different interconnected dimensions and the different periods of the SMC between its recommendation by the WHO and its scale-up in Burkina Faso, which supported observation and analysis of the processes studied (Fig. 2).

### Data analysis

Data analysis was performed using processual analysis. This method permits analyzing phenomena over time according to different vertical and horizontal levels of analysis and their interconnections [22]. The vertical level corresponds to multilevel analysis, looking at interdependencies between different levels to explain another level, and the horizontal level refers to sequential interdependencies between phenomena over time and corresponds to processual analysis. Using NVivo 11 software, we applied processual analysis to understand the interconnectedness processes underpinning SMC's sustainability and scaling up in time and space. In the results section, we have carefully distinguished between primary data (PD) and secondary data (SD) to better demonstrate the innovation's evolution and dynamics. We used two complementarity analysis strategies: a narrative strategy to describe and organize the chronological history of case events and a graphical or matrix strategy to present different elements succinctly and comprehensively [25]. In the analysis process, we focused on three elements: (1) different levels of analysis; (2) time, history, and action as interconnected; and (3) the link between the processes and the purposes. We used units and subunits of analysis presented in Fig. 2 to map the different contexts and processes throughout the intervention and the conceptual framework to interpret the results.

This study obtained ethical approvals from Burkina Faso's Health Research Ethics Committee (deliberation no 2018–3-033) and the Ethics Research Committee of the CHU of Quebec.

## Results

### Trends of SMC's implementation, scale-up and sustainability

**Fig. 3:**
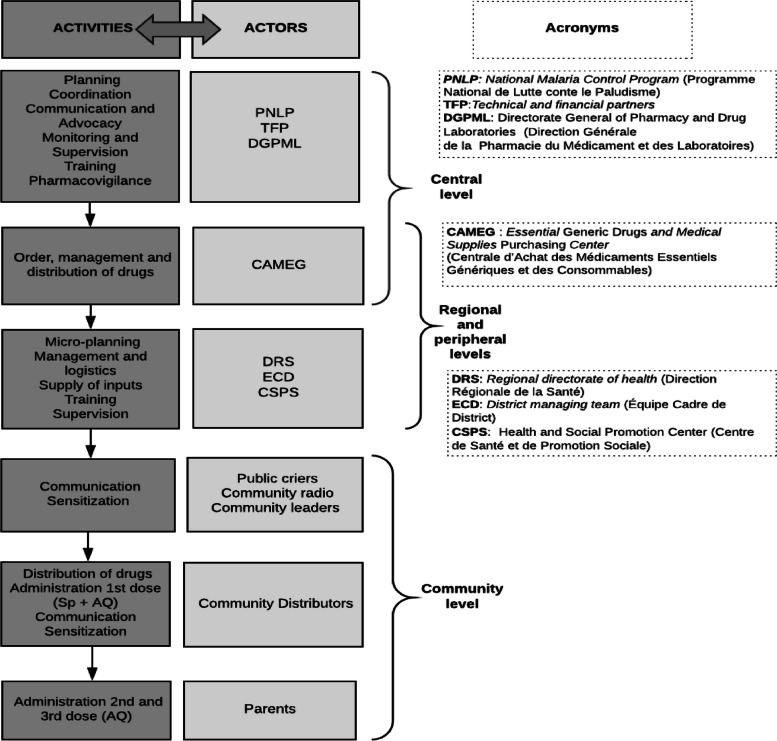
SMC implementation: activities and actors

For different central stakeholders, SMC was considered a relevant, new, and complex intervention in the fight against malaria in Burkina Faso. The managerial structure involved several types of partnerships and collaboration with different technical and financial partners (TFPs), organizations, health system actors, and community actors (Fig. 3). The SMC campaign was conducted on a centralized, tiered (or cascaded) approach throughout the health system; the activities of different stakeholders is described in Fig. 3. The PNLP [National Malaria Control Program] coordinated and monitored all activities and promoted SMC to policymakers and TFPs.

In 2014 and 2015, SMC was implemented as a pilot intervention in seven and then in 17 sanitary districts, respectively. A rapid scaling up started in 2016 to expand and replicate SMC in all 70 sanitary districts of the country. In 2018, the period of this study, 65 out of 70 districts nationwide were reached. The PNLP did not cover the remaining districts due to the absence of support from TFPs. The temporal progression of the scaling up analysis showed that TFPs accelerated the scale-up process, especially in widening geographical coverage and funding (Table 1). The arrival in 2015 of new TFPs with great funding opportunities, especially the World Bank and Malaria Consortium, mainly facilitated the scale-up decision. There was no plan for scaling up and sustainability at the central level; adding new districts depended on the arrival of new TFPs to maintain coverage progress.

*Based on the observation notes taken during some meetings, I noticed that the additional districts each year were based on the availability of funds.* (PD - Researcher)

**Table 1:** General picture of the evolution of SMC in Burkina Faso

Stage	Expected outcomes	Period	Decision	TFP (number of health districts covered)
***EMERGENCE*** **7** studies conducted between 2008 and 2011 in areas with high seasonal malaria transmission	-Prevents approximately 75% of all malaria episode and severe malaria episodes -May result in a decrease in child mortality of around 1 in 1,000 -Probably reduces the incidence of moderately severe anaemia	*2012*	**WHO recommendations in areas of high seasonal malaria transmission** *SP* + *AQ Administration for 4 months at regular monthly intervals*	
***IMPLEMENTATION in Burkina Faso*** Small scale pilot phase	-Annual reduction of 60% mortality related to malaria in children from 3–59 months -Reach 100% coverage -The accession of parents of target children to at least 90%	*2014*	**Implemented in 7 districts** *Deployment of SMC as a campaign for 4 days per month during: August, September, October, and November*	State of Burkina Faso ALIMA Terre des hommes
		*2015*	**Implemented in 17 districts** Start Access-SMC project lead by Malaria Consortium: expansion of SMC in the SAHEL region	Malaria consortium (11) World Bank (4) State / Unicef (2)
***SCALING-UP*** Progressive implementation in all the districts of the country		*2016*	**Implemented in 54 districts** Start Sahel Malaria and Neglected Tropical Diseases: cross-border coverage of SMC (Burkina Faso, Mali, and Niger)	Malaria consortium (29) World Bank (20) Global Fund (3) Malaria consortium/UNICEF (2)
		*2017*	**Implemented in 59 districts**	Malaria consortium (37) World Bank (18) Malaria consortium/UNICEF (2) PMI-JHPIEGO (2)
		*2018*	**Implemented in 65 districts**	Malaria consortium (18) World Bank (22) PMI-JHPIEGO (12) Global FUND (11) Global Fund/UNICEF (2)

The central-level actors perceived sustainability as an underlying element in scaling up. They considered it a process that will be achieved with maximum country coverage; its integration in the community and health system levels will take over with the maturity of the intervention. However, some participants considered that SMC will still need further implementation years to become a routine in the health system and communities. At the peripheral level, health professionals considered that SMC’s integration with other existing campaigns or their routine activities could even deteriorate the quality of the intervention: *You can integrate what is feasible. The activities [of SMC and routine activities in the health system] do not have the same duration. It is challenging. It will play on the quality of SMC* (SD—ECD TOUGAN).

Many participants at all levels viewed SMC as a top-down and standardized intervention that occurs only with higher-level instruction without the effective participation of all implementation levels.

### Scale-up and sustainability processes: the determinants

Data analysis revealed several interconnected elements that can positively or negatively influence the SMC sustainability and scaling up in Burkina Faso.

#### Effectiveness of SMC: real efficiency and its reputation for being effective

In 2018, there had been no national evaluation to determine the actual effectiveness of SMC after four years of implementation. The central-level actors have stated it was difficult under population-based implementation conditions to achieve the expected results of a 75% reduction in malaria incidence as announced by the WHO (Table 1). Based on the administrative data of sanitary districts in different years of implementation, the decrease in malaria incidence did not follow the same trend in all districts. For example, according to the SMC Implementation Synthesis Report (2016), the Boussé district showed a 59% reduction in 2014. In 2015, Ziniaré and Tougan districts had a 49% and 46% reduction, respectively. Thus, the absence of cyclical evaluation research on the intervention and an acceptable degree of decline in the effectiveness of the intervention during scaling up could have real consequences for the continuation of the intervention in the long term.

Despite the lack of evidence regarding effectiveness, SMC has a good reputation in health care settings and communities. Health professionals noted a brief decrease in the use of health centers during the SMC campaign; however, they questioned the long-term effectiveness of the SMC drugs. According to several participants, communities accepted SMC better than other mass immunization campaigns. The importance of malaria, which generates costs in money, time and mortality, especially among children, could explain this high acceptance and broad geographical coverage.

*SMC has helped us because, during the winter season, you can go to the health center with your child three or four times. However, from last year until now, I have had a child that did not exceed three years, that is, once I brought him to the hospital.* (SD - Mother BOULSA)

The positive reputation of SMC could negatively affect the caregivers' administration of the two doses of AQ. Indeed, according to regional and peripheral actors, caregivers did not always understand the pertinence of only targeting children aged three to 59 months when malaria affects everyone. There was uncertainty among various actors and documents about whether the second and third doses of the drug had been correctly administered by caregivers. This fact leads to lapses in effective drug administration, consequently affecting the drug's future effectiveness.

#### Inadequacies in follow-up and supervision

The follow-up and supervision of SMC were done at different hierarchical levels to enable the different actors (central, regional, and peripheral) to adjust the intervention and rectify certain shortcomings. However, some informants deplored the lack of integration of supervision activities between the different organizations and actors, which could affect the supervision efficiency and effectiveness.

*In the sanitary districts, we say, we must organize integrated supervision. What is happening? We take a vehicle, put 3 or 4 people, and then go to the health team. Everyone looks for their information, and then we take a report, and we say it is integrated supervision; it is not integrated supervision. (…) We have integrated the means to go for ineffective supervision. I say that integration, as we live it, is certainly not the best way, and it plays on the quality.* (PD - TFP)

On the other hand, actors encountered many challenges with follow-up and supervision because of the lack of qualified personnel, time, or resources. For example, head nurses have met several challenges to doing well in their supervision with community distributors because they did not receive enough fuel during the campaign, had many villages to cover, and had to attend to their routine activities. In addition, the supervision of community distributors with the children's caregivers was not mandatory. The ineffective administration of drugs has the risk of drug resistance development. For many actors at different levels, these deficiencies could hinder the quality and even equity of the intervention. Regarding equity, some participants complained that the supervision was not being carried out adequately in some inaccessible villages due to their distance from health centers, poor road conditions, and floods during the rainy season. Some TFPs wondered about the actual capacity of PNLP to ensure the expansion of the impact of the innovation across the country equitably.

*The PNLP cannot go to all the villages to check how it is going. Even the supervisor is not sure they reach all those in charge of administering. They only do sampling to see if it is okay. Often when supervisors go out, there are inaccessible areas; they do not even care what happens there.* (PD - TFP)

#### Human, material and knowledge resources: between the desire for integration and the realities of the verticality of SMC

Using human resources from various health systems represented an excellent way to potentially improve the SMC campaign. Several of these actors had experience and capacities in implementing mass campaigns. However, several actors at the peripheral and community levels complained about the extra workload with SMC, which was added to other campaigns during the year and various routine activities. They faced numerous challenges, such as insufficient staff and material resources and the mobility of human resources that limited their capacities in implementing SMC. Work overload can also affect the quality of the work done, especially by community distributors. Due to time constraints, some participants noted that community distributors did not sufficiently inform caregivers about drug use and, more broadly, health education on malaria prevention. According to many participants, several head nurses expressed the need for better training for community distributors, especially in interpersonal communication, to prevent drug resistance.

*This concern about drug resistance due to the lack of communication between community distributors and parents of children is present. Community distributors found they were too overloaded for the activity with minimal motivation. They had to do more than 50 distributions a day. During the campaign, they often give away the drug to parents and do not say anything. In our research, very few parents had comprehensive information.* (PD - Researcher)

Increasing the financial incentives was considered essential by several participants to ensure the sustainability of the actions carried out by health professionals and community distributors. However, some actors outside the health system (TFPs or research actors) considered financial incentives as a constraint to sustaining the intervention. For the latter, these incentives reinforce the idea that SMC is an independent activity that is not integrated into the practices of the health system and the communities. In contrast, ending financial incentives could hinder the continuity of the intervention or decrease the quality of implementation, as seen in this excerpt:

"*It is fine, we can want to help the population to have good health, we can do that, but there will be a difference between being motivated and not being motivated*" (PD – Head nurse).

#### SMC funding: between affordability and stabilization

Stakeholders saw funding as critical in SMC sustainability and scaling up processes. The TFPs largely supported the intervention, particularly the operational and input costs (medicines, logistical support, training, communication, and monitoring tools). Apart from their funding for the pilot phase (2014), the state contributed indirectly to SMC, notably through the human and material resources of the health system and through World Bank funding. Nevertheless, SMC drugs were free for communities, which is an opportunity that could encourage adherence and reinforce equity in the expansion of the intervention's impact.

Various central-level stakeholders considered SMC an affordable intervention because the capped cost at around $2.12 and $2.65 per child, per campaign, corresponds to an estimated monthly cost per child of about $0.85 (SD—Malaria Consortium, Saving Lives—SPC at Scale) or $1.00 (SD—Management Sciences for Health, 2016, April 28). In practice, lacking a cost estimate for SMC, the PNLP adjusted to funding at the district level but did not exceed the average cost-per-child threshold. This cost cap had positive and negative implications for the sustainability of the intervention and its expansion. Indeed, TFPs considered the cost of SMC per child, enabling them to support it financially. However, funding increased each year because it did not depend only on the cost per child but also on the geographical coverage (the addition of new districts and needs) and the annual target population growth. Thus, some partners with limited budgets decided to reduce their support, phase out the campaign in some districts, or not increase or decrease the number of districts covered in the following years (Table 1). Likewise, funding based on the cost per child negatively affected implementation. Several actors in the community and peripheral levels have voiced non-satisfaction with their needs (inadequate working materials for community distributors, low financial motivation) or the decrease in funding for specific activities (lack of dissemination of communication).

*Comparing the past year [2015], which was the beginning [in this district], we see that the communication activities were well managed. However, this year [2016], SMC communication has not been deleted, but donors did not fund their diffusion. This aspect remains relevant since it is complicated for communities to accept and understand the campaign when communities have not been well informed. (SD - ECD BOULSA)*

Stabilizing funding was an essential element in the continuation and scaling up of SMC. Indeed, most of TFPs' available funding expired in 2019–2020. Even though malaria eradication is an international concern, some partners encountered difficulties in mobilizing funds. Notwithstanding the arrival of new partners, such as Global Fund (Table 1), stakeholders had a sense of uncertainty about the sustainability of the scale-up. Several of them hoped for a practical commitment from the state to the direct funding of SMC that could enhance their appropriation of the intervention, which is essential to its sustainability.

*For sustainability, PTFs should empower the State (...) because usually, when the partners leave, it is over. There is no relay. If the State-owned the innovation, we know that at all levels, it will go. (SD - DRS FADA)*

#### Leadership and governance: between the desire for collaborative planning and the absence of inclusive governance

The PNLP tried to create synergy with the different actors involved in the SMC funding and implementation to ensure coherence throughout the intervention. Thus, the micro-planning was flexible and made at the peripheral level from the generic chronogram of the intervention and organizing post-campaign. However, collaborative planning faced challenges related to the partners, who often decided which activities would be funded and implemented in their districts. Furthermore, the multiple partners involved in the SMC did not work in coordination. Only those who had co-financed an area tried to pool their resources. For example, in the Boucle du Mouhoun Region (SD—*Bilan CPS 2017*), there was no harmonization of planning and practices between the district funded by JHPIEGO and the five districts funded by the World Bank.

Communities, including community distributors, were considered an essential pillar of SMC's governance. However, according to several participants and documents consulted, they played a passive role in decision-making. Their contribution was solely to maximize the adhesion of the population, the drug distribution and administration, and the dissemination of the prevention information related to malaria. According to some actors, this limitation of their involvement would not allow the incorporation of SMC into their routine in the long term.

*I will add especially about the empowerment of the population. Communities should be more involved in being actors in preventing their health rather than beneficiaries. They cannot protect themselves and ask for their needs. (PD - Researcher).*

In addition, poor governance related to the health system influenced the sustainability and extension of SMC. Indeed, in one district, the head nurses refused to organize the two passages of SMC in 2017 due to mismanagement of the financial and human resources at the peripheral and community levels, a lack of transparency, and corruption in mass campaigns. One of the nurse’s claims was the need to “*Stop the organization on the credit of mass treatment campaigns; transparency in campaign management*” (SD -Press article). Some actors considered the governance problem much more structural, and poor governance threatened SMC’s sustainability.

#### Simplification of a complex intervention: balance between harmonization and adaptation

Participants considered the SMC campaign a complex intervention. The central-level actors thought harmonization was necessary for SMC’s success through a uniform implementation in all districts by standardizing campaign dates and training, monitoring and supervision activities and tools, the number of community distributors, training, communication, and costs. However, many actors have thus seen harmonization as a hindrance to adapting innovation to the realities of the peripheral and community levels. For example, some head nurses criticized harmonizing the number of community distributors as unfavorable for some villages with certain geographical or population specificities that others do not have.

*It must be said that the number of community distributors was never enough in terms of sanitary areas to cover. Because there is not only the target as such to cover, but there are the villages too. There are distant concessions or hamlets of culture, and the PNLP does not consider that.* (SD- Head nurse BOULSA)

Nevertheless, peripheral and community actors have greatly appreciated some simplifications made in the harmonization process, particularly the suppression in 2017 of the registries that community distributors used to fill in their work and the change in the drug formula from a non-dispersible pill to a dispersible flavored pill. Actors perceived these changes as positively affecting the drug's administration and the implementation's quality.

## Discussion

This study aimed to understand the scaling and sustainability processes of SMC in Burkina Faso from 2014 to 2018. Several findings are consistent with those of previous studies. However, some discoveries are relevant for the empirical and theoretical development of the scaling up and sustainability areas; we highlight and discuss them in detail.

### Dependence on external funding and challenges of innovation ownership

This study shows that the rapid scale-up of SMC intervention depended mainly on external funding from multiple TFPs involved in the intervention. This support is an opportunity to stabilize and rapidly scale the innovation over a short period. However, withdrawing TFPs is a real risk to innovation sustainability since most funding comes from partners rather than the state. Several studies raised this risk and found that at the end of funding, the continuity of the innovation is entirely the responsibility of the government or local actors, who often need more financial resources [13, 26, 27] and the motivation to sustain continuation [28, 29]. In one study on outcome-based financing in Burkina Faso, at the end of the pilot project, several activities perceived as costly ceased or were reduced, such as supervision, meetings, and the monitoring of children [30].

The heavy reliance on TFPs funding emerges as a significant constraint for SMC ownership by the state and communities. We define ownership as the degree of control that recipient governments can exercise over the design and implementation of programs or projects, regardless of the objectives pursued [31]. SMC implementation should be oriented toward a people-centered approach and their actual capacities to use them. Our results show no long-term planning to prepare the various stakeholders to ensure SMC ownership through state and community empowerment. Implementers adopted a limited vision of the routinization activities to control the campaign to the detriment of capacity building by standardizing some routines (formation, supervision, collaborative planning) to ensure a rapid replication in the country. We suggest that routinization is not only a phenomenon that stabilizes innovation but also an element of change performed by agents [32]. Implementers should ensure SMC routinization at different contextual, individual, organizational, and institutional levels.

Furthermore, focusing uniquely on the scaling-up process does not ensure SMC institutionalization in the future. The absence of intensified actions to ensure the routinization of the intervention at the level of organizations or communities before starting the process of institutionalization could explain the observed failures of specific health policies [33–35], even if these interventions have proven their effectiveness during pilot projects. These observations support the theoretical proposition of Pluye et al. [17], whereby routinization is a stage that comes before institutionalization. If it is not well thought out and carried out considering different time frames, translating the pilot project into a health program or policy may not generate the expected results. In this sense, identifying critical actors at each stage of the scaling process and understanding their specific interests is essential to improving scaling effectiveness [36].

### The perennial challenge of collaboration between different partners

Our study and another [37] reveal that a sense of urgency and the drive to achieve quick results in innovation, combined with more diverse interests of multi-stakeholder partnership, may either engender too much or too little community involvement. The volatility of development assistance actualized through short-lived projects and the lack of coordination of the different actors involved are fundamental challenges for the effective and equitable extension of the impacts of an innovation [38]. The multiplicity of TFPs that initiate or support SMC scaling up at different territorial and institutional scales, without strong state leadership, contributes to the lack of a unique vision of the scale-up and sustainability processes and results. In this sense, scaling up is considered a systematic and uniform extension or replication of the innovation components, focusing on the geographical coverage and intensification of funding. This result is consistent with the usual considerations of health interventions scaling up in global health [19]. Sustainability is an outcome that arises "spontaneously" from the scaling up and implementation of the intervention. Implementers consider that the stages of the innovation are sequenced and linear [17, 39]; they do not plan the scale-up and sustainability, which rely heavily on the success of the implementation. All efforts (advocacy, resource mobilization) are oriented towards improving the SMC activities' geographic coverage, funding, and “technical” implementation. This way of doing things has been a limitation to making the necessary adaptations to the different SMC implementation contexts and periods. Studies have suggested that the vision of scaling up should be thought out and developed from the beginning of the interventions so that, if successful, the innovation can benefit the populations that need it [21, 40]. Thereby, creative funding strategies must be found in the medium and long term to sustain SMC. Also, long-term and predictable funding could allow the intervention to mature and have lasting impacts on children's health. Moreover, the coordination and interdependence of different stakeholders with a strong state leadership that aligns with the needs of communities are essential to maintain SMC funding.

### The verticality of the SMC campaign: structural challenges

Although the innovation needs to evolve in an institutional space to facilitate scaling up, our results show that the institutions, particularly the health and community systems, are failing in several respects, which may contribute to the deterioration of the intensification of the SMC impacts. Indeed, rather than creating a space for learning, which makes it possible to improve practices and ensure the viability of the innovation at different scales, it has been observed that innovation is often grafted into the "bureaucratic machine" of the state [41]. That is a significant challenge for sustainability, particularly for maintaining the quality of innovation and the continuity of specific components of innovation. For example, the results of this study make it possible to raise organizational problems within state systems, such as the lack of management or the uneven management of human resources, the insufficiency of financial and logistical resources, the hierarchical organization of the levels of actor, the consideration of innovations funded by partners as a "private" entity, the lack of effective participation of communities in the governance system, etc. These insufficiencies explain the difficulty of the actors evolving in these power systems to qualitatively continue certain vital activities of the innovations, such as training and monitoring supervision at the withdrawal of TFPs. This same observation is relevant for community systems that disseminate SMC innovation practices. For example, it seems legitimate to ask about the sustainability of training, monitoring, supervision, and financial motivation activities that concern community distributors involved in the SMC campaign. This questioning is vital since some studies have noted the challenges and obstacles that community health workers encounter in the community case management of malaria [42, 43]. These obstacles include a lack of stable funding, retention challenges, inadequate training and supervision, and parallel and concurrent interventions. Our study and another [44] carried out on SMC in Burkina Faso showed that the heavy workload of community distributors contributed to decreasing their performance in the effective administration of drugs and the transmission of information to caregivers. Therefore, by grafting a new intervention such as SMC into a "faulty" system without providing systemic solutions to existing problems, there is a risk of jeopardizing the sustainability of innovations or the quality of other existing interventions. In this way, several participants advocated for the possibility of developing drug resistance. Some studies highlight that SP/AQ drug resistance is not impossible in the Sahel [7, 45]. This was the case in the past with chloroquine in Africa [14]. Some recommendations emphasize that innovations should not be driven by vertical delivery approaches but by systemic approaches to maximize synergies between health systems and innovations. The integration of SMC in a holistic approach to fight malaria to improve the health of children is relevant [12]. To achieve this, the effectiveness of the health and community systems is essential.

### Interdependence of determinants: theoretical implications

Our results also suggest that internal and external components of SMC are interdependent and influence its continuity in time and space simultaneously. Furthermore, some determinants of sustainability or scale-up could be concurrent opportunities and challenges for the intervention, mostly when combined with contextual elements. These determinants are effectiveness, monitoring and supervision systems, resources (material, human), funding, leadership and governance, simplification, and adaptation. To illustrate the interdependency of these determinants, our results show that the availability and stability of resources (financial, human, material, knowledge) are favorable conditions for achieving the geographical coverage and impact objectives of the SMC. For these favorable conditions to be sustained, other elements and processes must be considered, including (1) the arrival of new flows of funding and their maintenance; (2) these flows result from the availability of resources allocated to malaria and, in particular, to SMC at the national and international levels; (3) state ownership of the intervention; (4) the stability of the country; (5) the proper functioning of the health system; (6) adhesion and participation of communities, caregivers, and community leaders; and (7) the effectiveness of SMC.

These results highlight that SMC characteristics or attributes alone cannot explain the elements influencing the sustainability and scale-up processes. For example, the fact that SMC is compatible with the values and norms of the organizations that support it (the state, TFPs, or communities) is necessary, but more research is needed to understand the influence of various determinants on sustainability and scaling up. It is also essential to consider the interaction between the *innovation structure* (degree of complexity, relevance, available inputs, etc.); the *characteristics of the organizations that support it* (norms, values, capacities, rules, etc.); the *macro-environment* (funding and global interest in malaria); the *meso* (bureaucratic norms, values, organizational capacities, structural realities of health and community systems, etc.); and the *micro* (issues of illiteracy, accessibility of villages, birth certificates of children, economic activities, etc.).

The effects of a determinant on the sustainability or scale-up are complex and impact the existence, scope, duration, and specific design aspects of interventions [46]. The importance lies in understanding the influence of scale dynamics in internal and external innovation environments during scale changing. Indeed, the successful implementation of integrated malaria-related interventions depends on implementing progressive and synergistic actions at all local, national, and international levels [47]. In a scaling up process, our study reveals that sustainability has a transversal dimension since the continuity of the intervention over time and space seems essential. To better understand the dynamic of change during the scaling up, it is crucial to consider temporalities to detect the existing dynamic relationship between actions, processes, and contexts.

### Limits

This study has some limitations. The findings are not necessarily generalizable beyond the case studied; however, we aimed to produce innovative empirical and theoretical knowledge to understand the case studied. In this way, the methodological procedures followed and the contextual factors that contribute to the patterns of our results were presented in detail to help the readers engage in a transfer of knowledge from this study to new situations. Nevertheless, some institutional or contextual changes in the innovation may produce different results than those presented here. To deal with this, we have given contextual details, and we are taking a reflexive approach in our scientific procedure that could also strengthen the reliability of our results and their transferability to other contexts [48]. Also, using secondary and primary data sources allowed us to triangulate our results and have an evolutionary vision of the intervention. In addition, the analysis period over four years of the SMC implementation in Burkina Faso could be limited in capturing the future of SMC. However, we have tried to link some processes to results by raising how certain events may negatively or positively influence the continuity of the intervention.

## Conclusion

This study makes empirical and theoretical contributions by using a systemic and processual analysis approach to capture the dynamics of sustainability and scaling up of SMC and the process and context of change. Our findings highlight the usefulness of systems thinking to consider all contexts (international, national, subnational, and local) to achieve large-scale improvements in the quality, equity, and effectiveness of global health interventions. Thus, using a conceptual framework inspired by the general systems theory is an added value to this study. Systems thinking allows us to go against linear and mechanical models that do not allow us to detect the interconnectedness between different parts and processes of the innovation and the existing exchanges between inputs and outputs that occurred during the innovation development. As a result, the systemic perspective has made it possible to observe and model emergent and dynamic behaviors in the innovation and the dynamics particular to sustainability and scaling-up processes. It could also be a step forward in studies focusing on sustainability and scaling up and an essential tool for implementers. The proposed framework posits sustainability and scale-up as dynamic, reflexive, and learning processes that begin with the conception of innovation. Thus, it remains crucial in the scale-up and sustainability processes to ensure the equilibrium of powers between stakeholders to ensure quality and equity in innovation processes. We recommend that future studies focus on the pathways to integrate SMC in the existing approach to fight malaria and in the organizational and community routines. Structural changes in the health system are fundamental for sustaining and expanding this type of intervention in the long term in Burkina Faso. For example, policymakers could create strategies for comprehensively strengthening health structures, particularly by enhancing their financial situations, to effectively implement preventive innovation types and be more autonomous in their management instead of dependent on TFPs or aid development.

### Supplementary Information

**Additional file 1.** Additional details on methods of data collection.

## Data Availability

The datasets generated and analyzed in this study are not publicly available due to their confidentiality and the fact that the participants had not consented to their words or opinions being shared publicly but can be obtained from the corresponding author upon reasonable request.

## Abbreviations

CAMEG: Centrale d’achat des médicaments essentiels génériques et des consommables [*Essential Generic Drugs and Medical Supplies Purchasing Center*]
CSPS: Centre de santé et de promotion sociale [*Health and social promotion center*]
DGPML: Direction générale de la pharmacie du médicament et des laboratoires [*Directorate General of Pharmacy and Drug Laboratories*]
DRS: Direction régionale de la santé [*Regional directorate of health*]
ECD: Équipe cadre de district [*District managing team*]
PNLP: Programme national de lutte contre le paludisme [*National Malaria Control Program*]
SMC: *Seasonal Malaria Chemoprevention*
TFPs: *Technical and financial partners*
WHO: *World Health Organization*
