# Supplementary material for: Using systems thinking to understand the scale-up and sustainability of health innovation: a case study of seasonal malaria chemoprevention processes in Burkina Faso
Source: BMC Public Health. 2023 Oct 2;23:1902. doi: 10.1186/s12889-023-16729-x (PMC10544612; doi:10.1186/s12889-023-16729-x)
Supplement: Supplementary file 1 — Additional file 1. Additional details on methods of data collection. [file 12889_2023_16729_MOESM1_ESM.docx]

***Additional details on methods of data collection***

| Data collection | Description | Participants/Documents |
| --- | --- | --- |
| Documentary method (n=141) | | |
| Secondary interview | Collected between late 2016 and early 2017 as part of ISMEA program | 66 individual interviews (in verbatim) collected in Boulsa, Fada, and Tougan districts Central level: *PNLP*, TFP*, PADS*, DGPML**  Regional level: *DRS**  Peripheral and community levels: *ECD*, Head Nurses, Community distributors, public criers, mothers of children* |
| Reports, scientific papers | Departmental and organization reports, implementation organization briefs, scientific papers (brief, field notes, reports, presentation, articles) | 37 documents |
| Press articles |  | 2 documents |
| Tools and registry | Registry, tools for training, monitoring and supervision, data management, communication, procurement, and administration of the drug | 36 documents |
| Individual interviews (N = 15) | | |
| Primary interviews | Collected by principal investigator | 15 individual interviews: Central level: *PNLP*, TFP* and affiliated organizations, PADS*, CAMEG**  Peripheral level (Sapone district): *District Chief Medical Officer and Head nurse* –  Local researchers: involved in SMC research |
| Peripheral observation (n=4) | | |
| Primary observation | Collected by the principal investigator | 4 documents |

*Note. The list of acronyms and abbreviations is available in fig. 3
